# Clinical and radiological manifestations associated with triple-negative breast cancer in women from northern Peru. A case-control study

**DOI:** 10.3332/ecancer.2024.1720

**Published:** 2024-06-27

**Authors:** Raúl Sandoval-Ato, Patricia Coral-Gonzales, Sebastian Coronel-Arias, Luisa Espinoza-Mantilla, Grace Terrones-Chaparro, Victor Serna-Alarcón

**Affiliations:** 1 Escuela de Posgrado, Facultad de Medicina, Universidad Privada Antenor Orrego, Trujillo 13008, Perú; 2 Unidad de Investigación Clínica, Scientia Clinical and Epidemiological Research Institute, Trujillo 13001, Perú; 3 Servicio de Radiodiagnóstico, Instituto Regional de Enfermedades Neoplásicas Norte, Trujillo 13008, Perú; 4 Escuela Profesional de Medicina, Facultad de Medicina, Universidad Privada Antenor Orrego, Trujillo 13008, Perú; a https://orcid.org/0000-0001-8666-7188; b https://orcid.org/0000-0002-8734-4687; c https://orcid.org/0000-0002-2607-7191; d https://orcid.org/0000-0002-5465-7775; e https://orcid.org/0000-0001-6938-1401; f https://orcid.org/0000-0002-9803-6217

**Keywords:** clinical features, mammography, ultrasound, triple-negative breast cancer

## Abstract

**Objective:**

Triple-negative breast cancer (TNBC) has an aggressive clinical behaviour, with advanced stages at initial diagnostic evaluation, early recurrences and poor survival, so the purpose was to determine the clinical and radiological manifestations associated with TNBC.

**Materials and methods:**

A case-control study in women diagnosed with breast cancer from January 2015 to August 2022 at the ‘Instituto Regional de Enfermedades Neoplásicas del Norte’. We classified cases (Triple Negative subtype) and controls (Luminal A, Luminal B and HER2) according to immunohistochemistry ical analysis. Bivariate and multivariate logistic regression models were used to calculate the odds ratio (OR) with their respective 95% confidence intervals (CIs).

**Results:**

The medical reports of 88 cases and 236 controls were reviewed. Cases were more likely to report pain (*p* = 0.001), nodules on ultrasound (*p* = 0.01) and mammography (*p* = 0.003), superior median size (*p* < 0.05), posterior enhancement (*p* = 0.001) and moderate density (*p* = 0.003). Multivariate analysis identified that TNBC was more likely to have a nodular type lesion by ultrasound (OR: 9.73, 95% CI: 1.10–86.16; *p* = 0.04), ultrasound lesion larger than 36 mm (OR: 4.99, 95% CI: 1.75–14.17; *p* = 0.003) and moderate density (OR: 3.83, 95% CI: 1.44–10.14; *p* = 0.007).

**Conclusion:**

There are particular clinical and imaging manifestations of TNBC, showing that radiological lesions that presented characteristics in ultrasound as nodular type lesions larger than 36 mm and in mammography moderate grade density, were associated with this subtype of breast tumours in a Peruvian population.

## Introduction

Triple-negative breast cancer (TNBC) is a tumour subtype defined by negative oestrogen receptors, progesterone and HER2 gene amplification. It has an aggressive clinical behaviour, with advanced stages at initial diagnostic evaluation, early recurrences and poor survival [1].

The prevalence of TNBC increases in young women under 40 years of age, with African or Hispanic ancestry [2]. Reproductive history becomes relevant by including multiparity and early age at first pregnancy as associated factors [3]. In premenopausal women, the prevalence of TNBC was higher [4]. The clinical factors most frequently associated with TNBC are overweight and obesity [5, 6], tumour size at diagnosis (mean 36 mm), ductal histological type, stage II and III at diagnosis and high histological grade compared to non-TNBC tumours [7]. There is also a high prevalence of first- or second-degree family history of breast or ovarian cancer in patients with this immunohistochemical subtype [8].

The atypical presentation characterised by distinct clinical features, rapid growth and heterogeneous density, decreases the chances for TNBC to be diagnosed by mammography or ultrasound in early stages [9, 10], delaying diagnosis with an impact on the survival of these patients [11]. Therefore, the objective was to determine the clinical and radiological manifestations associated with TNBC in women treated at the Regional Institute of Neoplastic Diseases in northern Peru.

## Material and methods

### Study design

A case-control study in women diagnosed with breast cancer during the period from January 2015 to August 2022 at the Instituto Regional de Enfermedades Neoplásicas del Norte ‘Dr. Luis Pinillos Ganoza’ - IREN Norte.

### Definition of cases and controls

The case group consisted of all patients with TNBC by immunohistochemistry (IHC); the control group included patients with luminal A, luminal B and HER2-positive breast cancer by IHC. In both groups, only patients who had results of their initial mammography and ultrasound studies were considered. All patients who did not have IHC results, patients who had undergone biopsies before their imaging studies, as well as patients with missing data on clinical-imaging variables, were excluded from the study.

### Data collection and variables

Permission was obtained from the institution’s authorities to access patients’ medical records. The data were collected using a virtual data collection form by six independent data entry clerks who were instructed in the correct collection of information between October and November 2022. This card included data on family and personal oncological history, socio-demographics, anthropometrics, hormone status, histological grade, clinical stage and description of lesions on physical examination. Imaging factors included a description of findings regarding size, breast density and the presence of sonic artefacts (shadow or posterior acoustic enhancement). The latter were reviewed according to the Breast Imaging Reporting and Data System lexicon and classification.

### Statistical analysis

Analyses were performed in the SPSS v.25 statistical software. Descriptive results were presented using absolute frequencies and percentages, measures of central tendency and dispersion. The association between clinical and imaging variables and TNBC was established using bivariate and multivariate logistic regression models. Odds ratios (ORs), and 95% confidence intervals (95% CIs) were calculated with statistical significance set at a value of less than 0.05 (*p*).

## Results

### Characteristics of the study population

**Figure 1.**
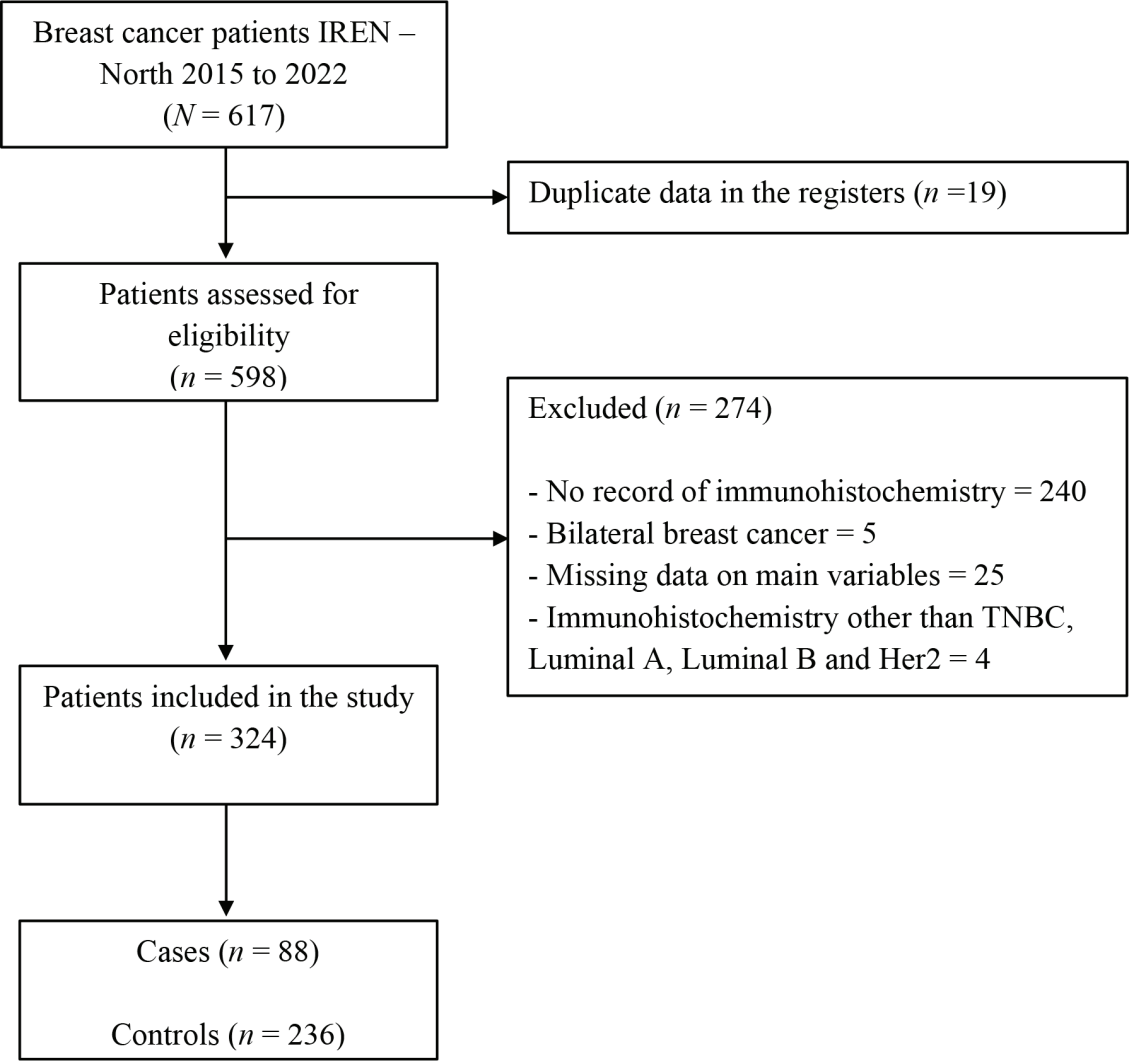
Patient inclusion flow for the study.

A total of 617 medical records of women diagnosed with breast cancer between 2015 and 2020 at the Regional Institute of Neoplastic Diseases ‘Dr. Luis Pinillos Ganoza’ – IREN Norte were evaluated, and 324 patients, 88 cases and 236 controls were included in the study because they met the selection criteria (Figure 1).

The distribution of baseline characteristics of the study groups is presented in Table 1. Age at diagnosis was similar in cases and controls (52.7 years versus 52.1 years; *p* = 0.38). Region of origin (Costa), overweight, postmenopausal hormonal status and clinical status II and III were the most common characteristics in cases and controls.

### Clinical examination findings associated with TNBC

On clinical examination, patients with triple-negative cancer more frequently reported pain compared to controls (59.4% versus 19.9%; *p* = 0.001).

Univariate regression analysis from clinical examination findings identified that breast pain was significantly associated with TNBC (omnibus test: ×2: 37.19; sensitivity 60%, specificity 80%), with a six-fold increase (OR: 6.00, 95% CI: 3.26–11.01) as opposed to women with other breast cancer subtypes (Table 2).

### Lesions diagnosed on ultrasound and mammography associated with TNBC

Among ultrasound-diagnosed lesions, a statistically significant difference in frequency of a nodule (91.7%), lesion size greater than or equal to 36 mm (47.3%), undefined or ill-defined margins (52.6%), posterior enhancement (44.1%) and posterior shadow (12.1%) was found among cases compared to controls. Bivariate regression analysis identified that lesions with posterior acoustic enhancement increase the likelihood of TNBC by 3.5-fold (OR: 3.55, 95% CI: 1.49–8.44, *p* = 0.004; Nagelkerke’s *R*^2^: 0.090). The finding of a nodular-type lesion increases the probability of TNBC sixfold (OR: 6.05, 95% CI: 1.29–28.43; *p* = 0.02, Nagelkerke’s *R*^2^: 0.168), when adjusted for posterior acoustic reinforcement of the lesion. A nodular lesion detected on ultrasound was shown to increase the probability of TNBC thirteenfold (OR: 13.37, 95% CI: 2.36–75.63; *p* = 0.02; Nagelkerke’s *R*^2^: 0.267), when adjusted for posterior acoustic enhancement and tumour size > 36 mm (Table 3).

The frequency of mammographically assessed lesions such as the presence of nodules (82.4%), a lesion greater than or equal to 21 mm (76.1%) and moderate density (70.0%) were found to be significantly higher among cases compared to controls. Bivariate regression analysis identified that lesions with moderate density increased the likelihood of TNBC up to sixfold (OR: 5.80, CI: 2.33–14.45; *p* < 0.001; Nagelkerke’s *R*^2^ : 0.174). Finding a lesion >21 mm on mammography increases the odds eightfold (OR: 8.50, 95% CI: 2.27–31.83; Nagelkerke’s *R*^2^ : 0.311), when adjusted for moderate lesion density. A nodular-type lesion detected on mammography was found to increase the probability of TNBC elevenfold (OR: 11.65, 95% CI: 1.40–96.56; *p* = 0.001; Nagelkerke’s *R*^2^ : 0.391) when adjusted for moderate lesion density and size >21 mm (Table 4).

It was identified that a nodular lesion detected by ultrasound is associated with a tenfold increased likelihood of TNBC (OR: 9.37, CI: 1.10–86.16), if this nodular lesion is matched to an ultrasound size greater than 36 mm and moderate density on mammography (Nagelkerke *R*-squared: 0.236; Omnibus test: ×2: 22.21; *p* < 0.001) (Table 5).

## Discussion

In Latin America, the frequency of TNBC in young women reaches 35%, with the highest rates reported in countries such as Peru and Mexico [9]. Worldwide, triple-negative tumours account for 12%–17% of all breast cancers, representing 24% of newly diagnosed breast neoplasms [12].

Detection by physical examination by the clinician, including clinical lesions found by the patient herself, has been reported as the most frequent method of detection of triple-negative breast tumours with a range of 68%–70.7% [13, 14]. The study of clinical features revealed that patients with TNBC were most frequently found to have a palpable mass at diagnosis, a common finding reported in the literature [15]. Also, the presence of pain on clinical examination represented a six-fold increase in the likelihood of presenting with this subtype of breast cancer. The presence of nipple retraction was found less frequently, findings corroborated by Long *et al* [16] who in their study reported that patients with the TNBC subtype presented less frequently with this same clinical sign when compared to the rest of the subtypes.

Histology revealed a highly undifferentiated grade in lesions compatible with TNBC. Previous studies have noted a significantly higher difference in the frequency of histological grade III in patients with TNBC compared to the other subtypes [17–19]. In Peru, a highly undifferentiated grade was found to be a feature consistently associated with TNBC with an increased prevalence of 70% of this finding [7].

The diagnostic approach by imaging studies has been a challenge for evaluators. To address this problem, the present investigation addressed the imaging characteristics that allow prediction, finding that, from lesions diagnosed by ultrasound, the presence of posterior acoustic enhancement and a lesion larger than 36 mm increased the probability of TNBC by up to five times, while the finding of a nodular lesion increases this probability by a factor of thirteen.

The presence of an ultrasound lesion compatible with a mass or nodule was significantly more frequently present in triple-negative breast tumours compared to luminal and HER2+ subtypes (86% versus 84% versus 68%) [20]. Lesion size has been a feature highlighted in previous studies, with a median size of 42.5 mm for this tumour subtype, compared to receptor-positive tumours [21]. Well-demarcated margins in 25% of TNBC and posterior acoustic enhancement are the most frequent presentations found on breast ultrasound [22, 23]. Positive enhancement is associated with tumour necrosis in triple-negative tumours, in contrast to other breast pathologies where its presence indicates benignity [24]. Considering that in our region breast characteristics are typically of increased density [8], attention to these findings on breast ultrasound would increase the diagnostic probability of this subtype of breast cancer, based on previous studies where ultrasound has been established as the ultrasound of choice in this subgroup of dense breasts [25]. 

On mammographic evaluation, this subtype of breast cancer is not associated with calcifications and irregular, spiculated margins, characteristic of luminal subtypes, because it does not usually present in situ stage due to its rapid growth [22]. TNBCs are evident on mammography as round, oval or lobulated masses, without architectural distortion, less likely to demonstrate features of malignancy [26]. Mammographic data collected in this investigation showed that moderate density increased the probability of finding a triple negative tumour sixfold, while lesions larger than 21 mm and the presence of nodules increased this probability tenfold and elevenfold. The reported frequency of masses or nodules varied from 49.0% to 85.0% between studies [20, 27, 28]. Density categorised as heterogeneous and elevated has been consistent findings, most frequently found in recipient-negative tumours [26, 27]. Lesion size in previously reported TNBCs is similar to our findings (mean 34.4 ± 15.7 mm) [29].

Despite these features found on each of the imaging tests, unlike other cancer subtypes, TNBC is diagnosed in approximately 19.6% of patients by mammography or ultrasound [13]. In the present study, an increased likelihood of TNBC was found in the presence of nodular lesions on ultrasound, ten times more; ultrasound lesions larger than 36 mm, five times more; and moderate density by mammography, up to four times more; data that are consistent with studies highlighting larger ultrasound tumour size and heterogeneous to high mammographic density [23]. 

Clinical, imaging and histopathological characterisation by subtypes is essential in the diagnostic and prognostic approach to patients [30]. The clinical usefulness of the associations presented are related to the ability of clinicians to have a high diagnostic suspicion of this entity and to help implement with the help of subsequent studies clinical-imaging risk models adapted to our population, generating a diagnosis without major delays represented by the component of health services, given the clinical aggressiveness and scarce therapeutic resources resulting in a poor prognosis of survival [31].

Since our study included data available from the last 6 years, the addition of information from later years would increase the accuracy of the observed results. Also related to this limitation, the database was obtained from a single institution and excluded about 44% of patients whose medical records were approached for eligibility due to a lack of breast cancer subtype characterisation. However, only those with complete IHC data were considered in the final analysis. Also, the significant results presented can be extrapolated to a population similar to the one included, which most frequently came from coastal areas of our region. Thus, these findings should be interpreted with caution for regions of other latitudes and altitudes due to different clinical characteristics and associated factors, and further studies should address the heterogeneity of our national population, with multilevel analysis disaggregated by geographic area being advantageous.

## Conclusion

In conclusion, the present case-control study showed that clinical and imaging manifestations are different in TNBC compared to the other subtypes. According to multivariate analysis, radiological lesions with ultrasound features such as nodular lesions larger than 36 mm and moderate grade density on mammography were associated with this subtype of breast tumours in a Peruvian population. Therefore, these features should be taken into account during breast cancer screening, given the worse prognosis of the triple-negative subtype.

## Conflicts of interest

The authors declare that there are no conflicts of interest.

## Funding

This study was self-funded by the authors.

## Informed consent

The use of informed consent was not necessary due to the type of research conducted.

## Ethics policy

The present research was based on the study of data from medical records from IREN-Norte. Approval was obtained from the Institutional Ethics Committee for access to this information. The confidentiality of the participants included was maintained. Data were used for research purposes only.

## Data availability

The database supporting the results of this study is available on request from the corresponding author.

## Supporting foundations

None.

## Author contributions

Patricia Coral-Gonzales: idea, study design, data collection, statistical analysis, data interpretation, writing the draft article, critical revision of the article and final approval of the version for publication.

Raúl Sandoval-Ato: idea, study design, data collection, statistical analysis, data interpretation, writing the draft article, critical revision of the article and final approval of the version for publication.

Grace N Terrones-Chaparro: data collection, data interpretation, drafting of the article, critical revision of the article.

Sebastian Coronel-Arias: idea, study design, data collection, statistical analysis, data interpretation, draft article writing, critical revision of the article and final approval of the version for publication.

Luisa Espinoza-Mantilla: data collection, data interpretation, drafting of the article and critical revision of the article.

Víctor Serna-Alarcón: idea, data collection, data interpretation, drafting of the article, critical revision of the article and final approval of the version for publication.

## Figures and Tables

**Table 1.** Clinical and imaging characteristics of the study groups.

	Total *n* (%)	Cases *n* (%)	Controls *n* (%)	*p*
Age (years) (*n* = 324)	51 (43 a 60)	52.7 ± 14.4	52.1 ± 12.5	0.38^§^
Schooling (*n* = 311)				
Illiterate	7 (2.3)	3 (3.7)	4 (1.7)	0.24^b^
Primary	96 (30.9)	28 (34.6)	68 (29.6)	
Secondary	141 (45.3)	34 (41.9)	107 (46.5)	
Technical Superior	8 (2.6)	4 (4.9)	4 (1.7)	
Higher University	59 (18.9)	12 (14.8)	47 (20.4)	
Region of origin (*n* = 324)				
Lima	6 (1.9)	1 (1.1)	5 (2.1)	0.17^b^
Coast (different from Lima)	200 (61.7)	58 (65.9)	142 (60.2)	
Sierra	95 (29.3)	25 (28.4)	70 (29.7)	
Rainforest	12 (3.7)	0 (0)	12 (5.1)	
Foreign	11 (3.4)	4 (4.6)	7 (2.9)	
BMI (*n* = 188)				
<18.5 kg/mt^2^	1 (0.5)	1 (1.8)	0 (0)	0.25^b^
18.5–24.9 kg/mt^2^	67 (35.6)	18 (32.7)	49 (36.8)	
25–29.9 kg/mt^2^	74 (39.4)	18 (32.7)	56 (42.1)	
30–34.9 kg/mt^2^	32 (17.0)	13 (23.7)	19 (14.3)	
35–39.9 kg/mt^2^	12 (6.4)	4 (7.3)	8 (6.0)	
≥40 kg/mt^2^	2 (1.1)	1 (1.8)	1 (0.8)	
Hormone status (*n* = 324)				
Premenopausal	102 (31.5)	31 (35.2)	71 (30.1)	0.38^a^
Postmenopausal	222 (68.5)	57 (64.8)	165 (69.9)	
Clinical examination				
Palpable clinical lesion (*n* = 317)	308 (97.2)	81 (95.3)	227 (97.8)	0.26^b^
Orange peel (*n* = 265)	66 (24.9)	20 (29.0)	46 (23.5)	0.36^a^
Nipple retraction (*n* = 265)	65 (24.5)	6 (8.7)	59 (30.1)	**0.001^a^**
Pain (*n* = 265)	80 (30.2)	41 (59.4)	39 (19.9)	**0.001^a^**
Telorrhage (*n* = 265)	3 (1.1)	0 (0)	3 (1.5)	0.57^b^
Skin oedema (*n* = 265)	14 (5.3)	3 (4.3)	11 (5.6)	1.00^b^
Skin erythema (*n* = 265)	10 (3.8)	2 (2.9)	8 (4.1)	1.00^b^
Ulceration (n = 265)	10 (3.8)	1 (1.5)	9 (4.6)	0.46^b^
Histological grade (*n* = 324)				
GI	7 (2.2)	1 (1.1)	6 (2.5)	**< 0.001^a^**
GII	172 (53.1)	29 (32.9)	143 (60.6)	
GIII	145 (44.8)	58 (66.0)	87 (36.9)	
Clinical stage (*n* = 322)				
I	22 (6.8)	5 (5.7)	17 (7.3)	0.07^a^
II	98 (30.4)	20 (22.7)	78 (33.3)	
III	159 (49.4)	54 (61.4)	105 (44.9)	
IV	43 (13.4)	9 (10.2)	34 (14.5)	
Family history of breast or ovarian cancer (*n* = 323)				
Yes	57 (17.65)	19 (21.8)	38 (16.1)	0.23^a^
No	266 (82.35)	68 (78.2)	198 (83.9)	
Family history of cancer other than breast or ovarian (*n* = 323)				
Gynaecological cancer	15 (4.6)	6 (6.9)	9 (3.8)	**0.02^a^**
Gastrointestinal cancer	33 (10.2)	6 (6.9)	27 (11.4)	
Cancer of another site	24 (7.5)	12 (13.8)	12 (5.1)	
No	251 (77.7)	63 (72.4)	188 (79.7)	
Ultrasound				
Nodules (*n* = 283)	231 (81.6)	66 (91.7)	165 (78.2)	**0.01^a^**
Size of the lesion (*n* = 276) *	24 (17 a 39)	32 (23 a 55)	22 (16 a 32)	**0.001^c^**
Subsequent reinforcement (*n* = 193)	44 (22.8)	15 (44.1)	29 (18.2)	**0.001^a^**
Back shade (*n* = 192)	54 (28.1)	4 (12.1)	50 (31.5)	**0.03^a^**
Mammography				
Nodules (*n* = 268)	184 (68.7)	61 (82.4)	123 (63.4)	**0.003^a^**
Size of the lesion (*n* = 212) *	25 (19 a 40)	30 (22 a 50)	25 (17 a 40)	**0.009^c^**
Breast density (*n* = 170)				
Baja	12 (7.1)	2 (6.7)	10 (7.1)	**0.003^b^**
Moderate	74 (43.5)	21 (70.0)	53 (37.9)	
High	84 (49.4)	7 (23.3)	77 (55.0)	

* Expressed as median and ranges.^§^ Student’s *t*-test for equal variances;^a^ Pearson’s Chi^2^ test;^b^ Fisher’s exact test;^c^ U-Mann Whitney test

**Table 2.** Clinical examination findings associated with TNBC.

	*ß*	SE	*p*	OR*	95% CI	
					Inferior	Top
Pain	1.792	0.310	0.000	6.000	3.267	11.018
Constant	−1.792	0.212	0.000	0.167		

* Binary logistic regression. Wald method

Variables not in the equation: palpable clinical lesion, orange peel skin, smoking, hormone status

**Table 3.** Ultrasound-diagnosed lesions associated with TNBC.

	*ß*	*p*	OR*	95% CI	
				Inferior	Top
Rear acoustic reinforcement	1.268	0.004	3.554	1.495	8.447
Constant	−1.574	0.000	0.207		
Rear acoustic reinforcement	1.407	0.002	4.083	1.645	10.136
Ultrasound nodules	1.801	0.022	6.056	1.290	28.431
Constant	−3.157	0.000	0.043		
Rear acoustic reinforcement	1.578	0.002	4.844	1.819	12.899
Ultrasound lesion > 36 mm	1.599	0.002	4.950	1.804	13.582
Ultrasound nodules	2.593	0.003	13.376	2.365	75.633
Constant	−4.447	0.000	0.012		

* Bivariate logistic regression. Method: Forward stepwise (likelihood ratio)

**Table 4.** Lesions diagnosed on mammography associated with TNBC.

	*ß*	*p*	OR*	95% CI	
				Inferior	Top
Moderate density by mammography	1.759	0.000	5.807	2.332	14.458
Constant	−2.211	0.000	0.110		
Moderate density by mammography	1.988	0.000	7.301	2.761	19.305
Mammographic lesion > 21 mm	2.140	0.001	8.502	2.270	31.835
Constant	−3.948	0.000	0.019		
Moderate density by mammography	1.881	0.000	6.558	2.380	18.069
Mammography nodules	2.456	0.023	11.659	1.408	96.566
Mammographic lesion > 21 mm	2.330	0.001	10.283	2.675	39.530
Constant	−6.187	0.000	0.002		

* Bivariate logistic regression. Method: Forward stepwise (likelihood ratio)

**Table 5.** Lesions diagnosed on ultrasound and mammography associated with TNBC.

	*ß*	*p*	OR*	95% C.I. for EXP(B)	
				Inferior	Top
Nodular type lesion by ultrasound	2.276	0.041	9.737	1.100	86.167
Ultrasound lesion >36 mm	1.608	0.003	4.991	1.757	14.174
Moderate density by mammography	1.344	0.007	3.834	1.449	10.140
Constant	−4.730	0.000	0.009		

* Bivariate logistic regression. Method: Forward stepwise (likelihood ratio)
